# A Family of Plasmodesmal Proteins with Receptor-Like Properties for Plant Viral Movement Proteins

**DOI:** 10.1371/journal.ppat.1001119

**Published:** 2010-09-23

**Authors:** Khalid Amari, Emmanuel Boutant, Christina Hofmann, Corinne Schmitt-Keichinger, Lourdes Fernandez-Calvino, Pascal Didier, Alexander Lerich, Jérome Mutterer, Carole L. Thomas, Manfred Heinlein, Yves Mély, Andrew J. Maule, Christophe Ritzenthaler

**Affiliations:** 1 Institut de Biologie Moléculaire des Plantes du CNRS, Université de Strasbourg, Strasbourg, France; 2 Department of Disease and Stress Biology, John Innes Centre, Norwich Research Park, Norwich, United Kingdom; 3 UMR 7213 CNRS, Biophotonique et Pharmacologie/Université de Strasbourg, Faculté de Pharmacie, Illkirch, France; University of Kentucky, United States of America

## Abstract

Plasmodesmata (PD) are essential but poorly understood structures in plant cell walls that provide symplastic continuity and intercellular communication pathways between adjacent cells and thus play fundamental roles in development and pathogenesis. Viruses encode movement proteins (MPs) that modify these tightly regulated pores to facilitate their spread from cell to cell. The most striking of these modifications is observed for groups of viruses whose MPs form tubules that assemble in PDs and through which virions are transported to neighbouring cells. The nature of the molecular interactions between viral MPs and PD components and their role in viral movement has remained essentially unknown. Here, we show that the family of PD-located proteins (PDLPs) promotes the movement of viruses that use tubule-guided movement by interacting redundantly with tubule-forming MPs within PDs. Genetic disruption of this interaction leads to reduced tubule formation, delayed infection and attenuated symptoms. Our results implicate PDLPs as PD proteins with receptor-like properties involved the assembly of viral MPs into tubules to promote viral movement.

## Author Summary

In plants, spreading virus infection occurs via small pores in the cell wall named plasmodesmata that connect adjacent cells. Two decades have passed since the first discovery of specific viral proteins (movement proteins; MP) that assist this process. However, the manner by which these proteins adapt plasmodesmata to allow the movement of relatively large viral structures remains largely unknown. Here, we show that a family of plasmodesmata-located proteins, called PDLPs, which are conserved amongst higher plants, specifically mediate this process. PDLPs bind classes of MP that assemble into tubules within plasmodesmata to promote the movement of entire virions. This class of MP occurs for a diverse range of plant virus genera and we show that representatives of these viruses have MPs that bind PDLPs. The importance of PDLPs in this process was shown when reduction in accumulation led to reduced tubule formation, delayed infection and attenuated symptoms. Altogether, our study supports a scenario whereby the PDLPs work together to support virus infection of plants and as such provide important mechanistic insights into the movement mechanism of plant viruses within their hosts.

## Introduction

Propagation of viruses in higher organisms depends upon cycles of virus uptake and egress. In animals, progeny virions leave the cell by budding from the plasma membrane (exocytosis), lysis of the cell, or communication through tunnelling nanotubes [1], [2], [3], [4]. In plants, viruses do not exit from cells but spread from cell to cell in the symplast through plasmodesmata (PDs) [5], [6], plasma-membrane-lined channels that bridge the cell wall to achieve symplastic continuity. PDs also contain a central axial membranous component, the desmotubule, derived from appressed endoplasmic reticulum (ER). Since PDs are tightly regulated, viruses encode movement proteins (MPs) to extend structurally and functionally the restrictions on molecular flux through the PD channel [7], [8], [9].

Viral MPs can be grouped into several broad classes based upon protein secondary structure predictions [10] or functional studies of the virus movement mechanism [11]. In the majority of cases, the MPs cause only subtle modifications to the overall structure of PDs, for example in the formation of fibrous substructures within the central PD cavities [12]. Some MPs, however, assemble into tubules that profoundly alter PD structure by displacing the desmotubule inside the PD, preserving only the integrity of the plasma membrane [13], [14], [15]. These tubules aid the transport of virus particles or viral ribo-nuclear complexes [16] into neighbouring cells (for review, see [13], [14]). Viruses encoding tubule-forming MPs include economically important pathogens, such as *Grapevine fanleaf virus* (GFLV), a member of the family *Secoviridae*. Members of the *Caulimoviridae*, *Badnaviridae*, *Tospoviridae*, *Ilarviridae* and *Bromoviridae* families are also representatives of this latter group.

Researchers have made progress to identify host components interacting with tubule forming MPs. First, studies performed with *Cowpea mosaic virus* (CPMV) MP indicate that a PD component, probably associated with the plasma membrane, could serve as specific interaction partners and provide the catalyst for ordered assembly of MPs into tubules to facilitate virus spread [17], [18], [19]. With *Cauliflower mosaic virus* (CaMV), a predicted Rab acceptor named MPI7 that interacts in yeast two hybrid has been identified and interaction correlated to the infectivity of MP mutants [20]. The cell plate specific syntaxin KNOLLE copurifies with the MP of GFLV expressed in tobacco BY-2 cells, but its function in viral movement remains to be determined [21]. The HSP70 cochaperone DnaJ and the non-cell autonomous protein At-4/1 interact with the MP (NSm protein) from *Tomato spotted wilt virus* [22], [23]. However, the mechanism employed by tubule-forming MPs to assemble into tubules within the PD so displacing the desmotubule and ultimately leading to the passage of virus particles, remains unknown.

We have recently identified a family of proteins (termed PDLP) that localizes specifically to PD. These type-I membrane proteins were shown to traffic along the secretory pathway to reach PDs and more specifically the plasma membrane lining the interior of PDs [24]. In this study, we have investigated in detail the involvement of PDLPs in virus movement. We provide genetic and cell biological evidence showing that PDLPs specifically interact with tubule forming MPs and collectively promote the cell-to-cell movement of virus employing tubule-guided mechanism. Our results identify PDLPs as PD proteins with receptors-like properties for tubule-forming MPs involved in virus movement and disease development.

## Results

### PDLPs are located at the base of tubules within modified plasmodesmata and specifically interact with the MP of GFLV

PDLP1-8, previously called PDLP1a-h, are type-I membrane proteins, located on the plasma membrane at PDs (Figure S1) [24]. To investigate whether PDLPs are important for assembly of tubules, we studied the GFLV MP (called 2B) owing to its ability to maintain assembly into tubules when fused to fluorescent proteins (such as GFP) and when expressed ectopically using heterologous expression vectors [21].

**Figure 1:**
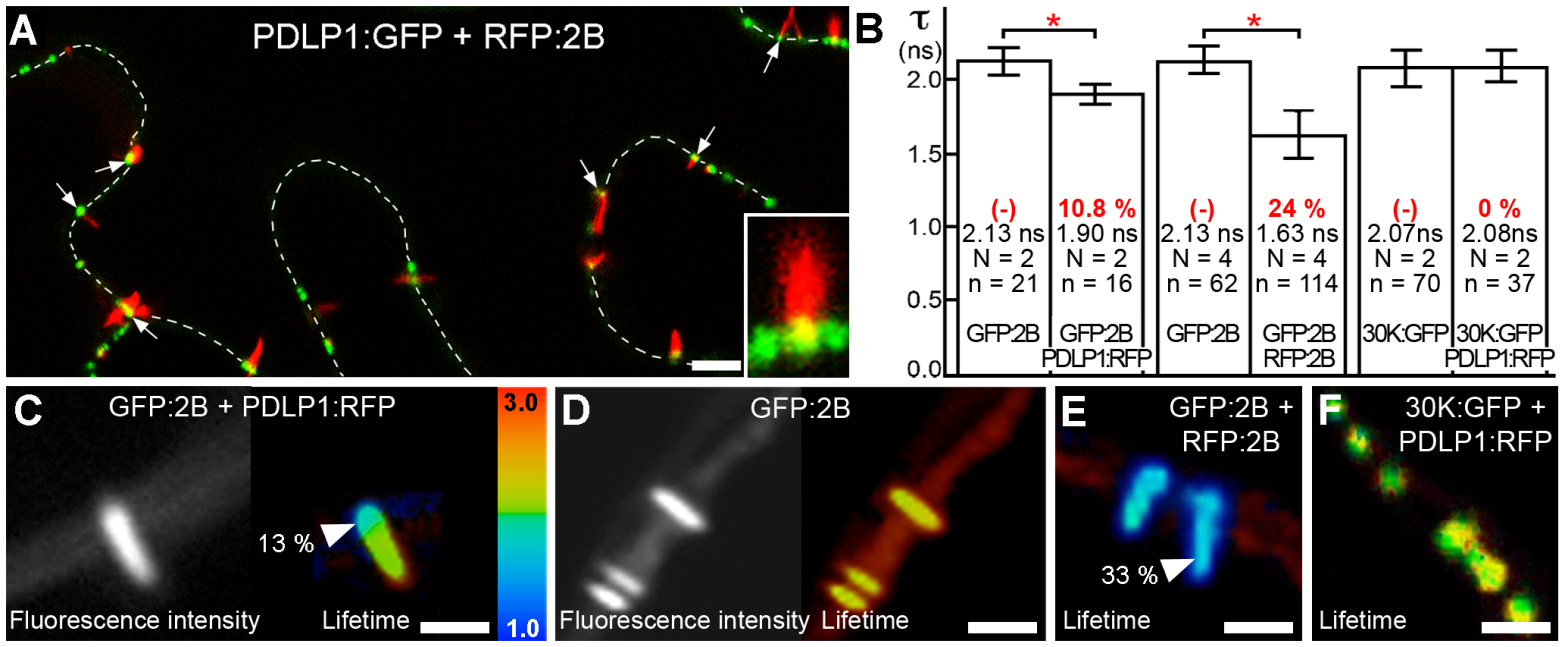
PDLP1 interacts with GFLV 2B at the base of the tubules. (**A**) RFP:2B tubule formation occurs on cell walls (dotted lines) at PDLP1:GFP-labeled foci. Colocalisation between PDLP1:GFP and RFP:2B is restricted to the base of the tubules (arrows; Inset, 4X mag.). (**B** to **F**) FRET-FLIM analyses. (**B**) Mean fluorescence lifetime (*τ*, ns) of GFP:2B over the length of the tubule when expressed alone or together with PDLP1:RFP (left bars), of GFP:2B over the length of the tubule when expressed alone or together with RFP:2B (central bars) and of 30K:GFP alone or together with PDLP1:RFP (right bars). Mean FRET values (percentage) are given in red. Significant differences (Student's *t* tests; *P*<0.05) are indicated with asterisks. Error bars  =  standard deviation. *n* is the number of measurements and N the number of independent experiments. (**C**, **D**) Fluorescence intensity (left) and lifetime images (right) of GFP:2B in the presence of PDLP1:RFP (**C**) and of GFP:2B alone (**D**). Note the change in lifetime restricted to the base of the tubule (**C**). (**E, F**) Fluorescence lifetime images of GFP:2B in the presence of RFP:2B (**E**) and of 30K:GFP in the presence of PDLP1:RFP (**F**). Note the color shift between (**D**) and (**E**). Fluorescence lifetime measurements are illustrated using the false color code shown in (**C**, right) ranging from 1 ns (blue) to 3 ns (orange). Donor and acceptor combinations and maximum FRET values were measured at sites identified by arrowheads. Bars  = 5 µm.

To test whether PDLP1 co-existed in PD modified by tubules, PDLP1:GFP and RFP:2B were transiently co-expressed in *N. benthamiana* following *Agrobacterium tumefaciens* infiltration. In agreement with previous report [21] MP-tubules always displayed similar orientation, having their base embedded in the cell wall and tip extending in the cytoplasm (Figure S1). Co-expression consistently showed that PDLP1 was located at the base of tubules within PDs (Figure 1A). The same observations were made when the fluorescent tags were reversed; i.e. transient expression of PDLP1:RFP and GFP:2B (Figure S2A). To assess whether co-location reflected a molecular interaction, analysis of direct protein-protein interactions using the FRET-FLIM technique [25] was used. This revealed a molecular association between PDLP1 and viral 2B at this location. Thus, using GFP:2B and PDLP1:RFP as FRET donor and acceptor, respectively, we observed a significant decrease in fluorescence lifetime for GFP:2B restricted to the base of the tubules, coinciding precisely with the location of PDLP1:RFP (Figure 1B, C). By contrast, when GFP:2B was expressed alone (Figure 1B, D) or together with RFP:2B (Figure 1B, E), no such variation in fluorescence lifetime along the whole tubule was observed (Figure 1D, E) although, in the latter case, an average of 24% of FRET was recorded (Figure 1B), reflecting the consequence of 2B homo-multimerization in tubules. This confirms experimentally the self-interaction of the tubule-forming MP in the tubule.

**Figure 2:**
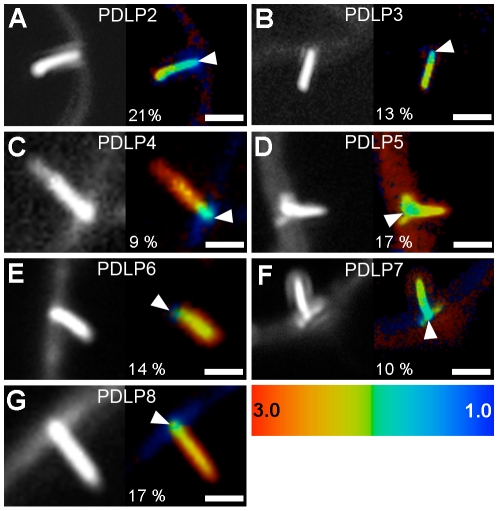
PDLPs collectively interact with GFLV 2B. Fluorescence intensity (left) and lifetime images (right) of GFP:2B in the presence of PDLP2:RFP (**A**), PDLP3:RFP (**B**), PDLP4:RFP (**C**), PDLP5:RFP (**D**), PDLP6:RFP (**E**), PDLP7:RFP (**F**) and PDLP8:RFP (**G).** Fluorescence intensity images are shown as grey scale pictures and lifetime images are represented using the false color code shown in the bottom right panel ranging from 1 ns (blue) to 3 ns (orange). Maximum FRET values (percentages) were measured at sites identified by arrowheads. Bars  = 5 µm.

To determine whether the interaction between 2B and PDLP1 was specific or simply resulted from the close proximity of over-accumulated proteins within PDs, FRET-FLIM measurements were also performed between PDLP1 and the MP from TMV (30K), whose mechanism of movement is not tubule guided [9], [26], [27], [28]; TMV 30K does however belong to the large ‘30K’ superfamily of MPs (Melcher 2000) and exhibits some functional overlap with tubule-forming MPs [16]. PDLP1:RFP and 30K:GFP were coexpressed in *N. benthamiana*. Although, as expected, the two proteins exhibited near perfect colocation [24] (Figure S2B), calculated lifetimes never varied significantly from those measured with 30K:GFP alone (Figure 1B, F), indicating that colocalization of proteins within PDs *per se* was not sufficient to generate FRET. Therefore, PDLP1 interacts directly and specifically with 2B at the base of the tubules. In Arabidopsis, the eight members of the PDLP family have varying patterns of tissue-specific expression, although all are expressed to some degree in leaf tissues and locate to PDs [24]. We found that, similar to PDLP1, all other PDLP isoforms gave significant FRET at the base of the tubules (Figure 2), indicating that they could also fulfil important functions in tubule formation and consequently in virus movement.

### PDLPs have redundant functions in 2B tubule formation

**Figure 3:**
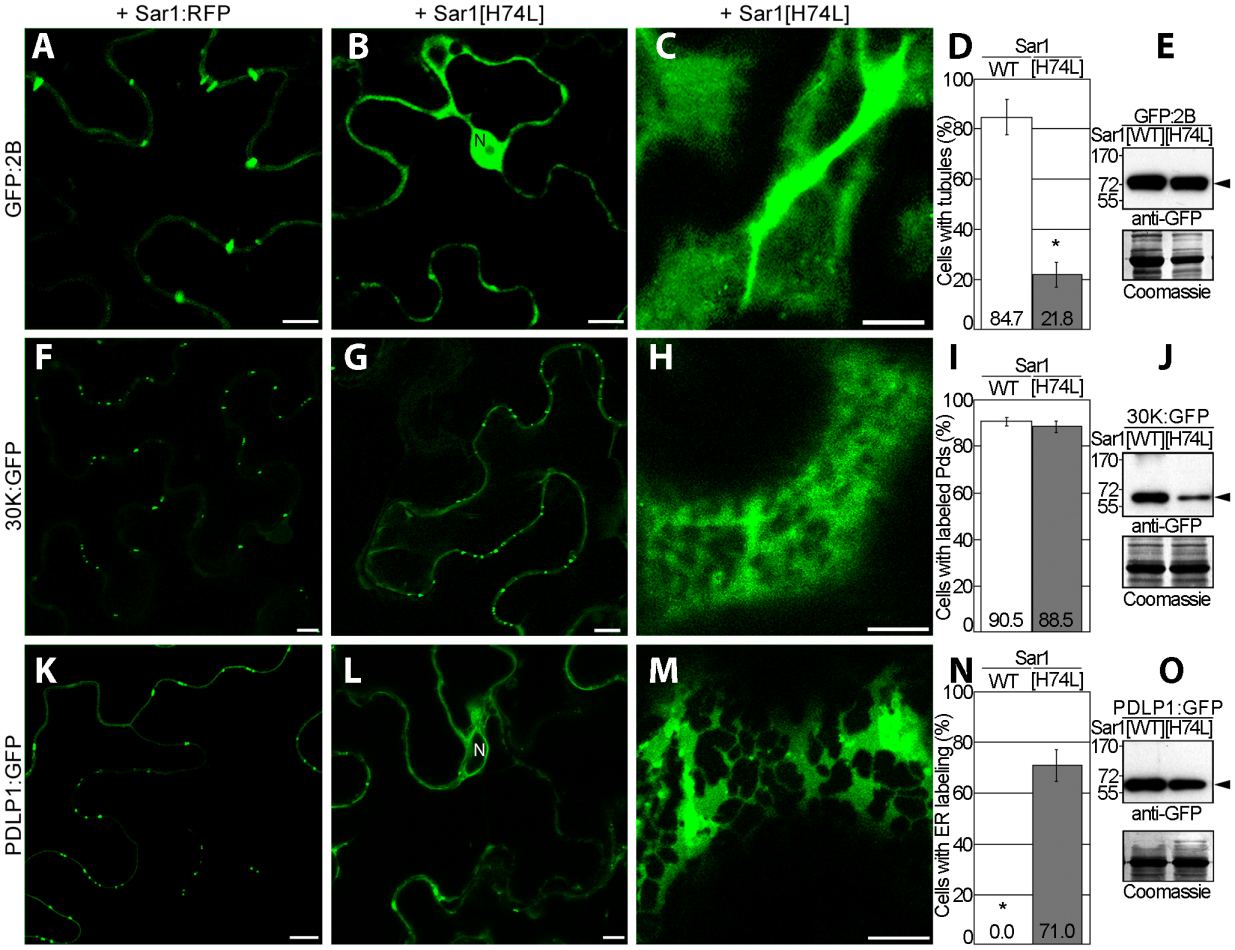
GFLV 2B is not a secretory cargo but inhibition of ER-export prevents tubule formation. Localization patterns and expression analyses related to GFP:2B (**A** to **E**), 30K:GFP (**F** to **J**) and PDLP1:GFP (**K** to **O**). Tagged proteins were coexpressed with either Sar1:RFP (**A**, **F**, **K**) or the secretion inhibitory mutant Sar1[H74L]:RFP (**B**, **C**, **G**, **H**, **L**, **M**), and location observed in median (left and central panels) or cortical (right panels) sections of leaf epidermal cells. Statistical analyses related to tubule formation efficiency by GFP:2B (**D**), plasmodesmal targeting of 30K:GFP (**I**) and ER-retention of PDLP1:GFP (**N**) upon coexpression with Sar1:RFP (white bars) or Sar1[H74L]:RFP (grey bars). Tubule formation efficiency is calculated as the ratio of cells containing tubules over the total number of fluorescent cells. Mean values are indicated in the histograms. Error bars, standard deviation. (**E**, **J**, **O**) Anti-GFP immunoblot (top) and Coomassie blue–stained gels (bottom) analyses of cells expressing GFP:2B (**E**), 30K:GFP (**J**) and PDLP1:GFP (**O**) together with Sar1:RFP or Sar1[H74L]:RFP. WT  =  wild type. Asterisks mark statistically significant differences between treated and mock-treated samples (*t*-test, *P<0.01*). N  =  nucleus. Arrowheads point at proteins of expected molecular mass. Scale bars  = 10 µm.

To test the hypothesis that PDLPs are needed for tubule formation, their accumulation at PD in *N. benthamiana* was prevented experimentally and the resulting effect on tubule formation was investigated. PDLPs are secretory cargoes transported to PDs via the endoplasmic reticulum (ER) [24]. Secretion can be inhibited by expression of a dominant negative mutant (Sar1[H74L]) of the Ras-like small GTPase Sar1 that specifically prevents ER export [24], [29]. Agro-infiltrated leaves transiently expressing GFP:2B, 30K:GFP, or PDLP1:GFP, were thus treated with Sar1[H74L]:RFP. For GFP:2B, the inhibitor drastically reduced tubule formation and resulted in GFP:2B accumulation in the cytosol and the nucleus (Figure 3B to E), whereas no effect on the accumulation of TMV 30K:GFP in PD was observed (Figure 3F to J). As expected, PDLP1 accumulated in the ER [24] (Figure 3L to O). The inhibition of targeting for 2B and PDLP1 was not observed when leaves were treated with the functional variant Sar1:RFP (Figure 3A, D, K and N). The absence of GFP:2B in the ER after treatment with Sar1[H74L] argues against 2B being a secretory cargo itself and against 2B carrying an independent PD targeting signal. The location of the PDLP-2B interaction only at the base of the tubule also argues against PDLP serving a chaperone function for 2B trafficking to PD. Precisely how 2B is trafficked to PD remains unclear.

**Figure 4:**
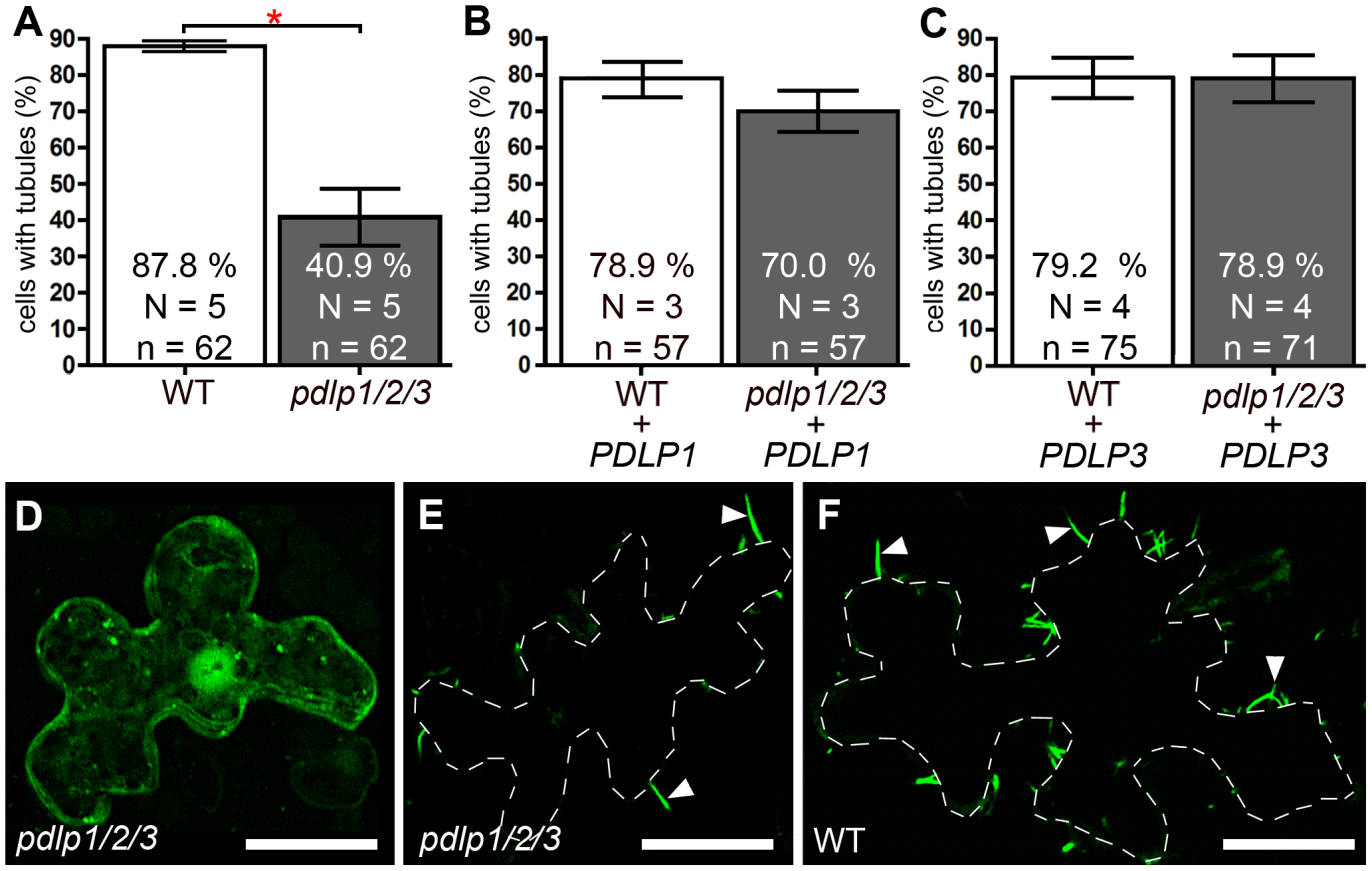
Genetic disruption of *PDLP* genes inhibits 2B tubule formation. (**A** to **C**) Tubule formation in WT and *pdlp1/2/3* mutants measured upon ectopic expression of GFP:2B alone (**A**) or together with *PDLP1* (**B**) or *PDLP3* (**C**). Statistically significant differences (Fisher exact test, *P*<0.001) are marked with asterisks. Error bars  =  standard errors. (**D** to **F**) Intracellular localisation of ectopically expressed GFP:2B in epidermal cells of the *pdlp1/2/3* mutant line (**D** and **E** show two cells from the same treatment, one without tubules (**D**) and one with reduced tubules (**E**)) and in cells of WT plants (**F**). **D** is a maximum intensity projection from 15 consecutive sections representing 6.75 µm; **E** and **F** are single optical sections. Bars  = 20 µm.

Similar to the physiological disruption of PDLP targeting upon inhibition of secretion, we anticipated that the genetic disruption of PDLP genes would also lead to reduced tubule formation *in vivo* and, consequently, to a limitation of virus spread and/or to enhanced disease resistance. However, the redundant character of the PDLP family presents particular challenges with respect to deleting this function in Arabidopsis. Double knock-out lines were tested for 2B-tubule formation *in vivo* following biolistic bombardment of constructs expressing GFP:2B into Arabidopsis epidermal cells. Homozygous *pdlp1-pdlp2* or *pdlp1-pdlp3* double mutants failed to show any significant difference from wild type plants (WT) in the efficiency of tubule formation. However, a triple mutant *pdlp1-pdlp2-pdlp3* (named *pdlp1/2/3* hereafter), which showed no obvious growth or developmental phenotype, exhibited a ∼46% reduction in the number of cells showing tubules when compared to WT plants (Figure 4A). In cells deprived of tubules, GFP:2B was distributed throughout the cytosol and nucleus (Figure 4D) similar to what was observed upon inhibition of ER export (Figure 3B, C). When detected, tubules were generally less numerous in cells from *pdlp1/2/3* plants compared to WT Arabidopsis (Figure 4E, F). The need for the triple *pdlp1/2/3* mutation to see the reduced-tubule phenotype suggested that these paralogues are acting redundantly. Therefore, we expected tubule formation to be restored in the *pdlp1/2/3* background if *GFP:2B* was co-bombarded with just one of the *PDLP* members and, indeed, co-bombardment with either *PDLP1* or *PDLP3* demonstrated a complete complementation of tubule formation in the mutant background (Figure 4B,C).

### PDLPs play important roles in helping tubule-guided virus movement

**Figure 5:**
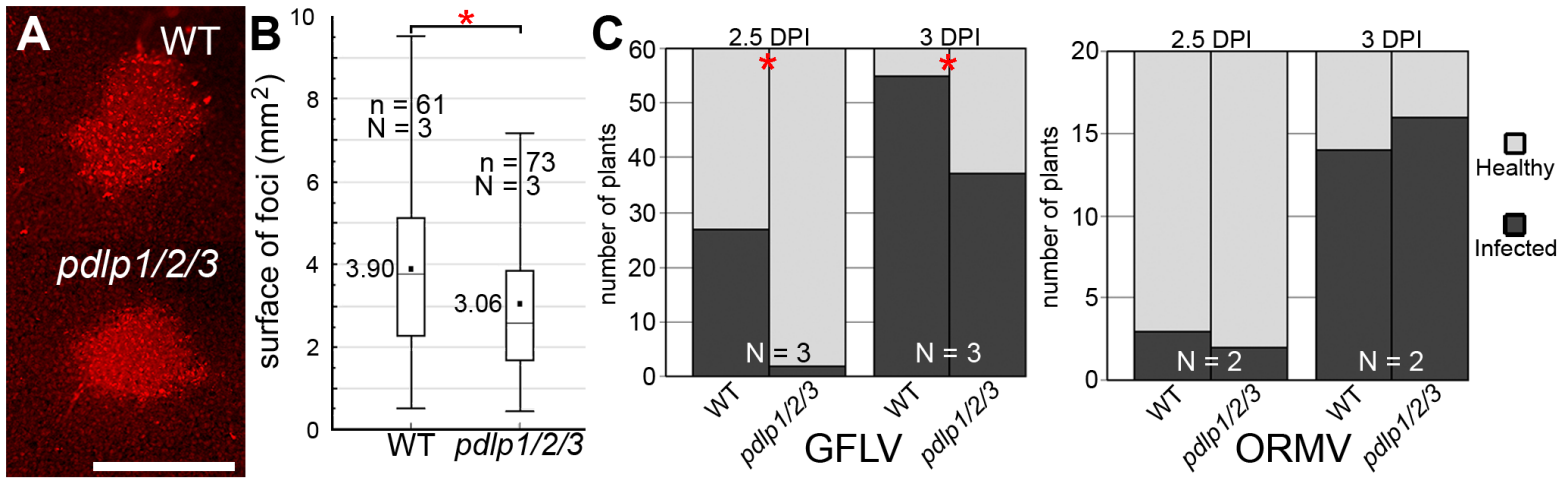
GFLV cell-to-cell and long distance movement is altered in *pdlp1/2/3* mutants. (**A**) Infection foci generated by GFLV:RFP on WT (top) and *pdlp1/2/3* (bottom) plants at 3 dpi. Scale bar  = 1 mm. (**B**) Corresponding box plot with whiskers from minimum to maximum of fluorescent foci size distribution. Calculated mean values are given for each graph. Significant differences (ANOVA; *P*<0.05) are indicated with asterisks. (**C**) Analysis of GFLV (left chart) and ORMV (right chart) long distance movement. Inoculated leaves were removed at 2.5 and 3 dpi and plants tested for systemic infection at 14 dpi. Light grey, healthy plants. Dark grey, infected plants. Significant differences (ANOVA, *P*<0.05) are indicated with asterisks. N  =  number of independent experiments. n  =  number of samples.

Arabidopsis serves as a susceptible experimental host for GFLV, giving rise to asymptomatic systemic infection. To test whether disruption of PDLP function and tubule formation had an impact on virus spread, GFLV was inoculated onto single leaves of WT and *pdlp1/2/3* plants, and local and systemic spread of the virus measured. A recombinant GFLV encoding TagRFP (GFLV:RFP) allowed the extent of local (cell-to-cell) spread to be measured from the area of fluorescent foci produced 3 days post-inoculation (dpi) (Figure 5A and Figure S3). Long distance movement of GFLV was assessed by removing inoculated leaves at various times after inoculation and scoring the ability of the infection to move systemically to distal organs. Significant differences in both local (Figure 5B) and systemic spread (Figure 5C) of infection were observed in the *pdlp1/2/3* mutant compared to WT plants. Thus, a 22% reduction in mean surface area of infection foci (Figure 5B) and an approximately 12 h delay in long distance movement (Figure 5C, left chart) were recorded in *pdlp1/2/3* versus WT plants. Under identical experimental conditions, no difference in systemic viral spread was observed if plants were challenged with the Arabidopsis–infecting *Oil-seed rape mosaic virus* (ORMV) (Figure 5C, right chart), a close relative of TMV whose cell-to-cell movement is tubule-independent [30], [31].

**Figure 6:**
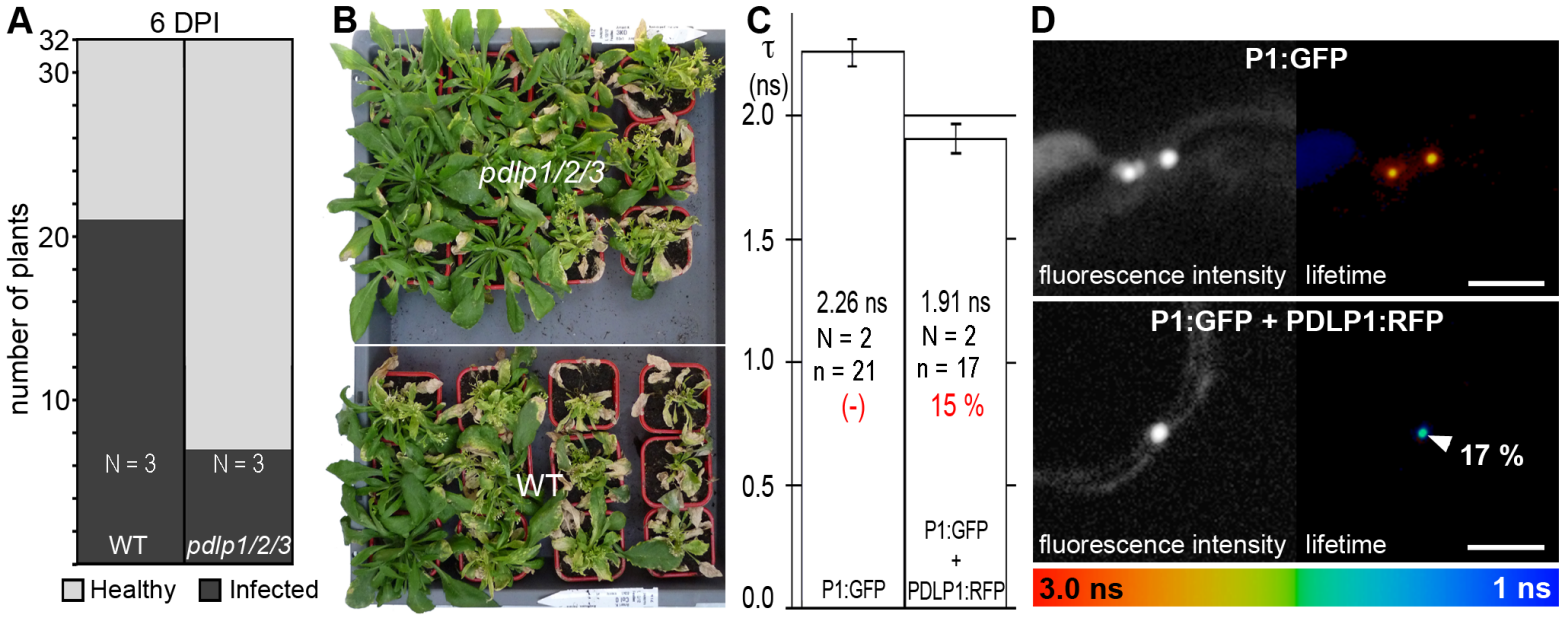
PDLPs are important contributors to CaMV movement *in planta*. (**A**) Analysis of CaMV long distance movement in WT and *pdlp1/2/3* plants. Inoculated leaves were removed at 6 dpi and plants tested for infection at 21 dpi. Light grey, healthy plants. Dark grey, infected plants. The treatments were significantly different at *P*<0.05 (ANOVA); N  =  number of independent experiments. (**B**) Symptoms observed on *pdlp1/2/3* (upper) and WT (lower) plants at 21 dpi. Plants were sorted by symptom severity from top left to bottom right (**C**) Fluorescence lifetime (τ, ns) measured for P1:GFP when expressed alone (left bar) or together with PDLP1:RFP (right bar). Mean FRET values (percentage) are given in red. The treatments were significantly different at *P*<0.05 (Student's *t-*test). (**D**) Fluorescence intensity (left) and lifetime images (right) of P1:GFP alone (top) and P1:GFP in the presence of PDLP1:RFP (bottom). Fluorescence intensity images are shown as grey scale pictures and lifetime images are represented using the false color code shown in the bottom panel ranging from 1 ns (blue) to 3 ns (orange). Donor and acceptor combinations are given in all panels. FRET value (percentage) was measured at site identified by arrowhead. Note: Although P1:GFP is targeted to PD, unlike GFLV GFP:2B, it is unable to form tubules unless supplemented with unfused P1 [35]. Error bars  =  standard deviation. n  =  number of measurements. N  =  number of independent experiments. Bars  = 5 µm.

Since GFLV is one representative amongst several groups of viruses that use tubules [13], [14], we asked whether the impact of *PDLP* disruption on infection would apply to another evolutionarily distinct virus, such as *Cauliflower mosaic virus* (CaMV) [32], [33], [34]. Although the long-distance spread of CaMV was slower than that of GFLV or ORMV, systemic invasion was similarly delayed in the *pdlp1/2/3* mutant when compared to WT plants (Figure 6A), and overall milder symptoms were observed in the *pdlp1/2/3* mutant (Figure 6B). In line with the involvement of PDLP1 in CaMV movement, PDLP1:RFP and CaMV MP:GFP (P1:GFP) colocalized within PDs (Figure S2C); P1:GFP does not form tubules when expressed in the absence of untagged P1 [35]. Significant FRET could be measured by FLIM, reflecting an interaction between P1:GFP and PDLP1:RFP within PDs (Figure 6C, D).

**Figure 7:**
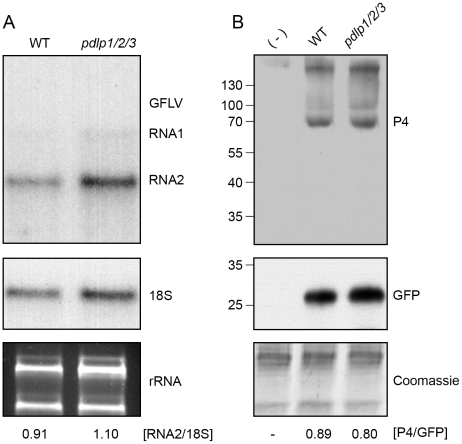
Genetic disruption of multiple *PDLP* genes does not affect GFLV and CaMV replication. (**A**) Detection of progeny GFLV RNA1 and RNA2 (top), 18S ribosomal RNA (middle) and total rRNA (bottom) from WT (left) and *pdlp1/2/3* (right) protoplasts, 72 hours post-transfection. GFLV vRNAs and 18S ribosomal RNA were detected by northern blot using radiolabeled probes. rRNA were detected after ethidium bromide staining. Ratios of RNA2 over 18S signal intensities are indicated below the respective lanes. (**B**) Detection of CaMV P4 capsid protein (top), GFP (transfection control, middle) and Coomassie blue stained proteins (bottom) from CaMV-infected WT and *pdlp1/2/3* protoplasts. (-) refers to healthy Arabidopsis protoplasts. Ratios of P4 over GFP signal intensities are given below the respective lanes.

In principle, some of the differences observed in the timing of cell-to-cell and systemic movement could be attributed to varying efficiencies of replication within individual cells. To test this possibility, protoplasts isolated from WT and *pdlp1/2/3* Arabidopsis plants were transfected with GFLV and CaMV, and viral levels subsequently assayed by northern analysis or by immuno-blotting (Figure 7). No significant variation in the accumulation of GFLV RNAs and CaMV proteins was observed in protoplasts from *pdlp1/2/3* and WT genotypes (Figure 7). This result shows that virus cell-to-cell movement rather than replication is affected in the *pdlp1/2/3* line.

## Discussion

GFLV and CaMV are distantly related viruses employing tubules to achieve virus movement. Here we show that the PDLP family of proteins interacts specifically within PD with the MPs of GFLV and CaMV. Although located in the same subcellular environment, a similar interaction was never observed for the MP of TMV, suggesting specific functions of PDLPs in tubule biogenesis and virus movement. In support of our hypothesis, the inhibition of trafficking of PDLP to PD through interference with ER export strongly reduced tubule assembly within PDs and led to the accumulation of the GFLV 2B in the cytoplasm. Similarly, *pdlp1/2/3* defects reduced GFLV local and systemic infection and attenuated CaMV systemic spread and disease. These mutations had no impact on virus replication or spread of ORMV, indicating a specific relationship between the MPs of these viruses and the host proteins. Based on these results, it is worth speculating that PDLPs could serve as PD-located proteins with receptor-like properties for tubule forming MPs and thereby catalyze their assembly into tubules to allow virus spread.

PDLPs are type-I trans-membrane proteins located on the PM at PD with a receptor-like domain located in the apoplast and only a short C-terminal tail in the cytoplasmic domain. The precise function of these proteins is not known except that ligand binding to the N-terminal domain and signalling into the PD space to modify PD flux has been proposed [24]. It seems unlikely that PDLPs arose specifically as necessary factors for virus movement, especially as the major protein domain is spatially separated from MP in the symplast, but more that tubule forming viruses have evolved the ability to exploit constituent PD proteins. The ability of GFLV 2B to interact directly with all the members of the PDLP family provides the virus with multiple opportunities to promote tubule assembly and virus movement through PDs and also a selective advantage against this step being a target for the evolution of natural resistance. The PDLP members have diverse tissue-specific patterns of expression potentially giving virus access to many tissue environments.

PDLPs have a cytosolic C-terminal domain ranging from 5 to 19 amino acids with poor sequence conservation that is unlikely to promote specific interaction with MP [24]. GFLV and CPMV MPs, however, are membrane-embedded proteins with the potential to interact with the trans-membrane domain of PDLPs within the membrane environment [19], [21]. This interaction could occur with PDLP *in situ* at PD or on the cytoplasmic face of the ER en route to PD. All these proteins were affected by the chemical and protein inhibitors of the COPII-mediated transport pathway ([19], [21], [24], this work), raising the possibility that PDLP could chaperone MPs to PD. However, the interaction of 2B with PDLPs only at the base of the tubule and the absence of retention of 2B in the ER upon inhibition of ER export tends to argue against a co-chaperone model and in favour of specific interactions occurring at PD.

To assess the relevance of these findings beyond our initial observations made for GFLV 2B, we selected the CaMV MP. *Nepovirus* and *Caulimovirus* MPs both occur within the tubule-forming subgroup of the ‘30K’ superfamily of MPs but show little direct sequence similarity [10]. In addition, caulimovirus MP tubules are wider [36], [37] to accommodate the 50 nm CaMV particles and mediate the transport of the encapsidated DNA genome as opposed to the RNA genome of nepoviruses. Since PDLPs interact directly with P1 and play important functions also in CaMV movement, we would speculate that they may be universal PD-located targets for tubule forming MPs. Although the full scope of this function remains to be tested, the behaviour of GFLV 2B, CaMV MP and *Cowpea mosaic virus* (CPMV) MP in cells treated with BFA shows interesting similarities. Hence, CPMV MP and CaMV MP tubules were inhibited in the presence of BFA and, as for GFLV 2B, BFA treatment leads to the redistribution of the MPs to the cytosol [19], [21], [38] and not to the ER or the BFA compartment, a behaviour otherwise expected for secretory cargoes such as PDLPs ([24] and this work). Although one cannot totally exclude the contribution of other yet unidentified host secretory proteins in tubule assembly, inhibition of tubule formation is therefore likely the indirect consequence of reduced PDLP accumulation within PDs due to arrest of secretion. Since PDLP homologues are widely distributed in plants [24] we can also suggest that they may provide necessary movement functions in a variety of host backgrounds.

To date, a number of host proteins have been shown to interact with viral MPs. Only a limited number of those locate to PDs and even less has been shown to regulate virus movement [6], [27]. Remorin, class 1 RGPs (Reversibly Glycosylated Polypeptides) and calreticulin are particular for their capacity to inhibit virus movement and to simultaneously locate to PDs [39], [40], [41]. They act as negative regulators of virus movement possibly via partial PD occlusion [41]. Thus an increase in calreticulin, RGPs or remorin levels leads to decreased efficiency of virus movement [39], [40], [41] and *vice versa* [39]. So far, this makes PDLPs unique as plasmodesmal proteins with agonist function in virus movement since a decrease in PDLP expression is correlated to increased viral resistance.

In conclusion, PDLPs specifically and collectively interact with MPs that have the capacity to assemble into tubules within PDs. We provide genetic and physiological evidence to support the redundant function of PDLPs in the tubule assembly process and movement of distantly related viruses that employ tubule-guided mechanism. Our results provide evidence to support a scenario whereby PDLPs act as proteins with specific receptor-like properties required for tubule-forming MPs assembly at plasmodesmata and thereby cell to cell movement of viruses employing tubule-guided movement. Future work will aim at deciphering the nature of the interactions between tubule forming MPs and PDLPs through structural approaches.

## Materials and Methods

### Plant material

All analyses were performed on *Nicotiana benthamiana* or *Arabidopsis thaliana* (ecotype Columbia). All plants were maintained in growth chambers under 10–12/12–14 light/dark cycles, 21/18°C day/night temperature and approximately 70% humidity. The *pdlp1-pdlp2* and *pdlp1-pdlp3* double mutant lines were as described [24] and used to generated the *pdlp1-pdlp2-pdlp3* (*pdlp1/2/3* hereafter) mutant line by standard genetic crosses. Homozygous mutant genotypes were confirmed by allele-specific PCR assays after two generations. All comparisons between WT and *pdlp1/2/3* plants were performed on plants of same age and size and maintained at all stage of development under identical growth conditions.

### Cloning and vectors

All PDLP and 2B binary vectors were generated by GATEWAY cloning according to the manufacturer's instructions (Invitrogen). p35S::PDLP(1 to 8):RFP binary vectors were generated using the entry vectors described previously [24] and the destination vectors pH7RWG2 [42]. The open reading frame encoding the 2B of GFLV was PCR-amplified from pTA7002-MP [21] and recombined into pDonR/Zeo (Invitrogen). To generate p35S::GFP:2B and p35S::RFP:2B which encode EGFP or mRFP1 N-terminally fused to the 2B open reading frame, respectively, the destination vectors pK7WGF2 and pH7WGR2 were used [42]. Inserts of expression vectors were verified by DNA sequencing. The infectious RNA1 clone pMV13 [43] and RNA2 clone pVecP2 [44] of GFLV were used to generate GFLV:RFP in which protein 2A is fused to TagRFP [45]. To do so, mutagenic oligonuclotides and inverse PCR was used to introduce an in-frame *Xba*I site into pVecP2 immediately upstream of the codons corresponding to the Cys/Ala cleavage site separating protein 2A and 2B frame. TagRFP open reading frame was then PCR-amplified in order to introduce *Xba*I sites within the 5′ and 3′ extremities and also 18 extranucleotides to restore a 2A/2B maturation site between the TagRFP and 2B sequences. All other binary vectors were described elsewhere: p35S::PDLP1:GFP, p35S::Sar1:RFP, p35S::Sar1[H74L]:RFP, p35S::RFP-ER [24], pB7-MP:GFP expressing the 30K MP of TMV fused C terminally to fluorescent protein under control of the 35S promoter [46], p35S::P1:GFP (kindly provided by L. Stavolone, CNR, Instituto di Virologia Vegetale, Bari, Italy). All primers and oligonucleotide sequences are available upon request.

### Transient expression of proteins by agro-infiltration

Electro-competent agrobacteria (strain LBA4404.pBBR1MCS-5.virGN54D) [47] were used for transient expression as described earlier [46].

### Viruses and infection assays

GFLV viral RNA1 and recombinant RNA2 transcripts were inoculated first on *Chenopodium quinoa* leaves. Infected systemic leaves were collected 14 dpi. Sap extracts (1 g tissue/2 mL 25 mM sodium phosphate buffer pH 7) were used to inoculate *C. quinoa* to be used for virus purification. GFLV-F13 and GFLV-2A:RFP were purified from infected *C. quinoa* plants as described before [48] and adjusted to 50 ng/µl in 25 mM sodium phosphate buffer pH 7 for inoculations.

To monitor cell-to-cell movement, fully expanded leaves from 8 weeks old Arabidopsis rosettes were inoculated with 150 ng/leaf of purified GFLV:RFP and observed at 3 DPI under appropriate wavelength with a Leica MacroFluo equipped with the apochromatically corrected zoom system Z16 APO, a 5X objective and a DFC 360FX camera. All pictures were taken under identical illumination and exposure conditions to allow comparisons. A double blind procedure was followed for the manual delimitation of fluorescent foci. Size (area) of fluorescent foci was then calculated using Image J (Rasband, W.S., NIH, Bethesda, MD, USA, http://rsb.info.nih.gov/ij/, 1997–2009) and statistical analysis (ANOVA) performed from three independent experiments.

To monitor long distance movement, two leaves from 6 weeks-old Arabidopsis rosettes were inoculated by gentle rubbing with 150 ng/leaf of purified GFLV or ORMV and celite. Inoculated leaves were removed after 2, 2.5, 3 and 4 days post-inoculation (dpi). Due to the absence of symptoms in *A. thaliana,* the presence of GFLV in distal organs (roots) was assessed at 14 dpi by DAS-ELISA using standard procedures [49]. For ORMV, systemic infection was assessed by the presence of symptoms at 21 DPI and confirmed by DAS-ELISA. For CaMV, we followed the same procedure as described above with the exception that plants were 5 weeks old and 2 leaves per plant inoculated each with 1 mg of pCaMV-GFP [50]. Due to slower multiplication, the two inoculated leaves were removed at 6 dpi and systemic infection assessed by the presence of symptoms at 21 dpi and confirmed by PCR analysis of viral DNA as previously described [51].

### Tubule formation assays

Tubule formation by GFP:2B in Arabidopsis leaf epidermal cells was determined following particle bombardment essentially as described previously [24]. Tubule formation, counted as the number of bombarded cells with tubules relative to the total number of target cells (shown by RFPer or PDLP1.RFP), was determined at 48 h post-bombardment by confocal microscopy. Statistical analyses (Fisher Exact tests) were performed using Graph Prism software 4.0 (GraphPad software, San Diego, CA).

### Inhibitor studies

To analyse the effect of ER-export on tubule formation, *N. benthamiana* transiently co-expressing GFP:2B and Sar1:RFP or the mutant Sar1[H74L]:RFP were produced as described earlier [24]. PDLP1:GFP and 30K:GFP were used in the same experiment as secretion-dependent and -independent plasmodesmal marker controls, respectively [24], [52]. The effects of Sar1[H74L] on tubule formation was assessed by determining the percentage of fluorescent cells that show tubules, using CLSM. A total of 9×100 cells were analysed in three independent experiments. From the absolute number of fluorescent cells, mean percentage values were calculated.

### Immunoblot analysis

*N. benthamiana* leaf disks of equal size were collected and total proteins extracted in Laemmli buffer, resolved by SDS-PAGE and transferred by electroblotting onto a polyvinylidene difluoride membrane (Immobilon-P; Millipore). Membranes were probed with affinity-purified GFLV 2B-specific antibodies [53] raised in rabbits and diluted 1∶10000; polyclonal anti-30K (reactive against TMV-30K residues 209–222) antibodies raised in rabbits [54] used at 1∶4000 dilution; and monoclonal anti-GFP (ClonTech) antibodies raised in mouse and diluted 1∶5000. Anti-rabbit IgG whole antibodies (Kirkegaard and Perry laboratories KPL) or anti-mouse IgG horseradish peroxidase-linked whole antibodies (Invitrogen) were used as secondary antibodies and were detected with Lumi-light Plus Western Blotting Substrate detection reagents (Roche). Equal loading was assessed after staining of membranes with colloidal Coomassie blue.

### Protoplast transfection and analysis

Arabidopsis protoplasts were prepared, transfected, and cultured essentially as described [55]. Protoplasts were adjusted to 1.5×10^6^ cells/ml and 0.3 ml used for transfection with either 7 µg of purified GFLV or 100 µg of pCaMV-GFP plasmid DNA. After treatment with PEG for 30 min, the protoplasts were cultured for 72 h at 25°C under constant illumination at 22°C, harvested and further processed for immunoblot and nucleic acid analysis. Total RNA extraction and northern blot analysis of *A. thaliana* protoplasts was as described earlier for *C. quinoa* protoplasts [56] except that probes were radiolabeled by incorporation of [α-^32^P]UTP. Ethidium bromide staining of total RNA agarose gel and hybridization with 18S RNA-specific probe [57] radiolabelled with [α-^32^P]dCTP were used to confirm equal loading. RNA fragments specific for the GFLV RNA1 and RNA2 were 7,300 and 3,400 nt, respectively. Total proteins were extracted using the phenol/ammonium acetate/methanol method as described earlier [58] and final protein concentration measured at 280 nm using a Nanodrop 2000 spectrophotometer. For each sample, 25 µg of protein was loaded on a 12.5% SDS-PAGE and processed as described above (*Immunoblot analysis).* Membranes were probed with CaMV P4-specific polyclonal antibodies diluted 1∶20000 (kindly provided by Mario Keller, IBMP, Strasbourg) and monoclonal anti-GFP diluted 1∶5000 (ClonTech).

### Fluorescence Lifetime Imaging Microscopy (FLIM)

FLIM studies were performed as previously described [46].

### Statistical analyses

Statistical evaluations were performed with the R software using ANOVA, Student's *t-*test or Fisher exact test where appropriate. All ANOVA tests were followed by the post-hoc Tukey-Kammer test.

## Supporting Information

Figure S1 Compared with a normal PD (left), MP-tubules (containing virions) displace the desmotubule (De) while the plasma membrane (PM) in which PDLPs are inserted is retained. Note: the tubule is polar, projecting into the neighbouring cell. CW : cell wall. MP : movement protein. ER : endoplasmic reticulum.
(7.08 MB TIF)

Figure S2 PDLP1 location in relation to GFLV, TMV and CaMV MPs. (**A**) Transient co-expression of PDLP1:RFP with 2B:GFP (**B**) PDLP1:GFP with 30K:RFP and (**C**) PDLP1:RFP with P1:GFP in *N. benthamiana* observed in leaf epidermal cells. Leaves were co-agro-infiltrated with the different constructs and observed at 2 dpi using CLSM. Note: tubules do not form in P1:GFP expressing cells as a consequence of GFP fusion [35]. Scale bars : 5 µm (A) 10 µm (B,C).
(6.64 MB TIF)

Figure S3 Detection of GFLV:RFP in infected Arabidopsis leaves. Upper panels, examples of GFLV:RFP-induced fluorescent foci on inoculated leaves. Lower panels, signal detected on systemic (non inoculated), Arabidopsis leaves.
(5.36 MB TIF)
