# Supplementary figures and images for: A Family of Plasmodesmal Proteins with Receptor-Like Properties for Plant Viral Movement Proteins
Source: PLoS Pathog. 2010 Sep 23;6(9):e1001119. doi: 10.1371/journal.ppat.1001119 (PMC2944810; doi:10.1371/journal.ppat.1001119)

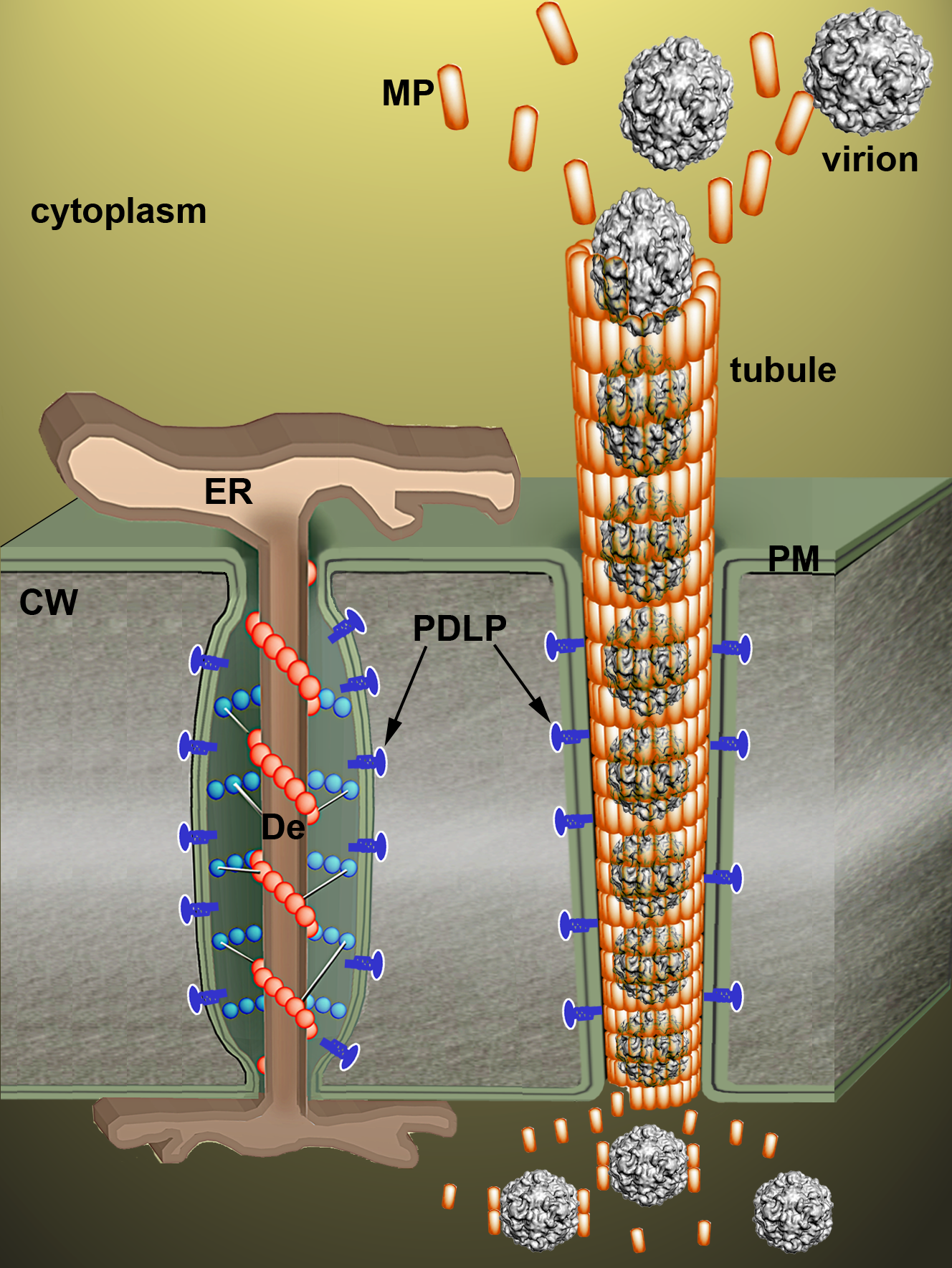

Supplement: Figure S1 — Compared with a normal PD (left), MP-tubules (containing virions) displace the desmotubule (De) while the plasma membrane (PM) in which PDLPs are inserted is retained. Note: the tubule is polar, projecting into the neighbouring cell. CW : cell wall. MP : movement protein. ER : endoplasmic reticulum. (7.08 MB TIF) [file ppat.1001119.s001.tif]

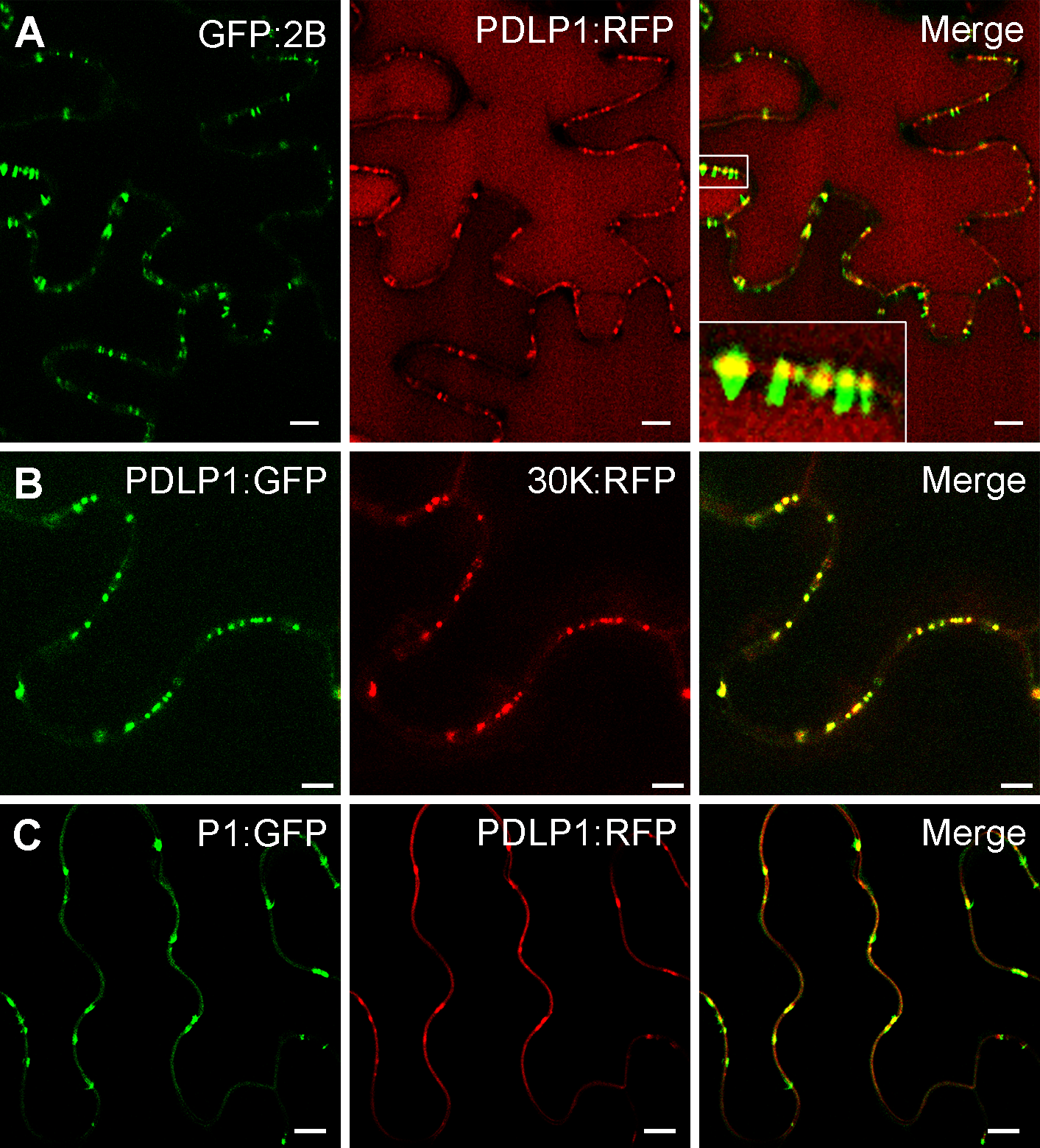

Supplement: Figure S2 — PDLP1 location in relation to GFLV, TMV and CaMV MPs. (A) Transient co-expression of PDLP1:RFP with 2B:GFP (B) PDLP1:GFP with 30K:RFP and (C) PDLP1:RFP with P1:GFP in N. benthamiana observed in leaf epidermal cells. Leaves were co-agro-infiltrated with the different constructs and observed at 2 dpi using CLSM. Note: tubules do not form in P1:GFP expressing cells as a consequence of GFP fusion [35]. Scale bars : 5 µm (A) 10 µm (B,C). (6.64 MB TIF) [file ppat.1001119.s002.tif]

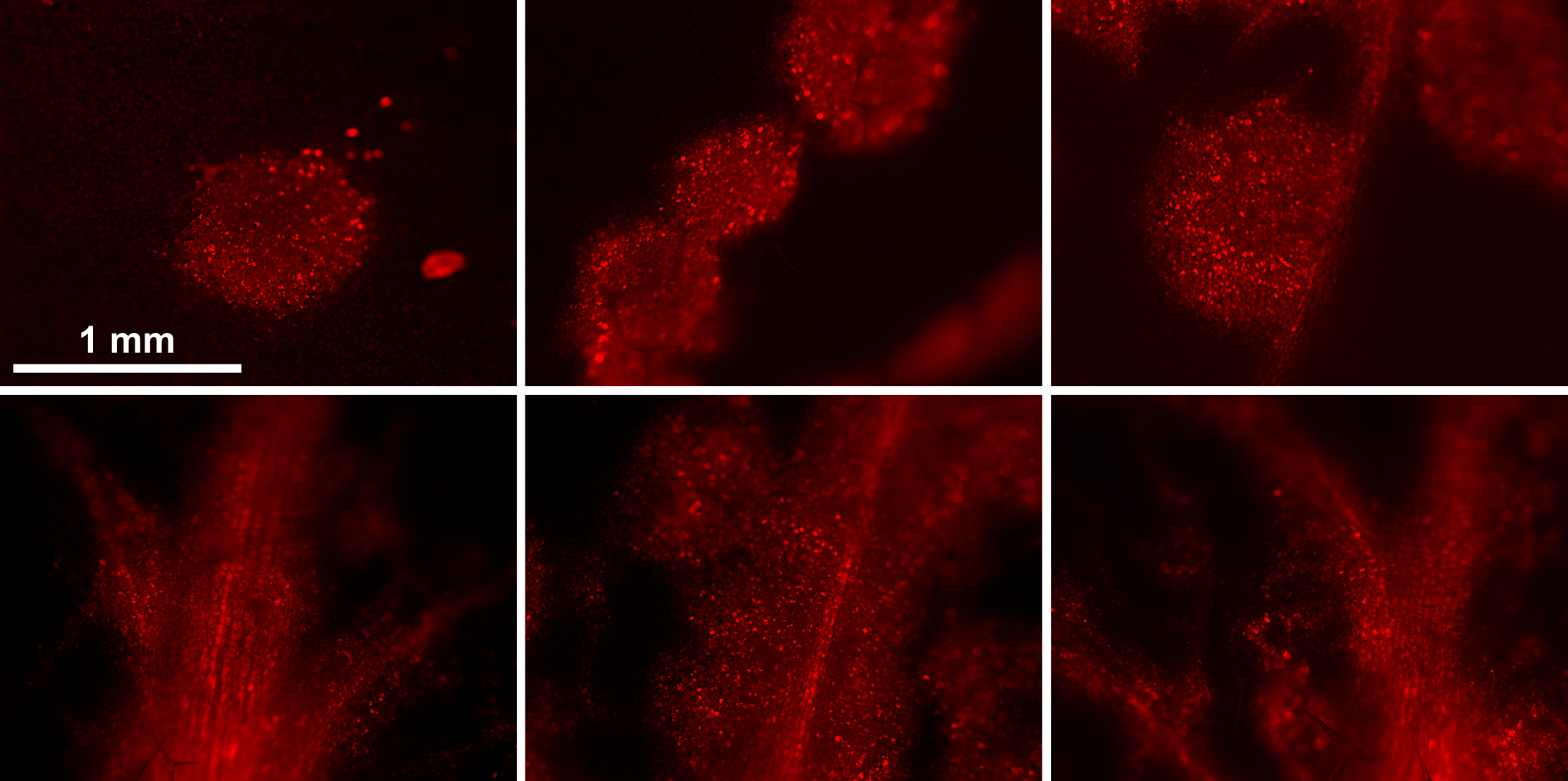

Supplement: Figure S3 — Detection of GFLV:RFP in infected Arabidopsis leaves. Upper panels, examples of GFLV:RFP-induced fluorescent foci on inoculated leaves. Lower panels, signal detected on systemic (non inoculated), Arabidopsis leaves. (5.36 MB TIF) [file ppat.1001119.s003.tif]
